# Temporal Trends of Linear Speed and Change of Direction Performance in Italian Children

**DOI:** 10.5114/jhk/189745

**Published:** 2024-12-06

**Authors:** Matteo Vandoni, Alessandro Gatti, Vittoria Carnevale Pellino, Caterina Cavallo, Agnese Pirazzi, Matteo Giuriato, Nicola Lovecchio

**Affiliations:** 1 Laboratory of Adapted Motor Activity (LAMA), Department of Public Health, Experimental Medicine and Forensic Science, University of Pavia, Pavia, Italy.; 2 National PhD Programme in One Health approaches to infectious diseases and life science research, Department of Public Health, Experimental and Forensic Medicine, University of Pavia, Pavia, Italy.; 3 Department of Sport and Exercise Science, LUNEX International University of Health, Exercise and Sports, Differdange, Luxembourg.; 4 Department of Human and Social Sciences, University of Bergamo, Bergamo, Italy.

**Keywords:** sprint, speed, physical fitness, exercise

## Abstract

Children tend to enjoy high-intensity activities that involve both linear speed (LS) and change of direction (COD), essential for sports performance. However, the results of trends in LS and COD have not been consistent over time. Therefore, our study aimed to investigate the temporal trends in LS and COD performance among 11–13 year old Italian children/preadolescents over 25 years, while minimizing the effect of anthropometric characteristics. A total of 3884 students were recruited between 1990 and 2010 and performed 4 x 5-m shuttle run, 30-m sprint, and 60-m speed tests. A weighted linear regression was performed to analyze the overall temporal trends in BMI-adjusted speed tests. The results showed an increase in mean 4 x 5-m shuttle run time, indicating a decrease in COD ability, while LS performance remained relatively stable over time. These trends were consistent across genders and ages. Our study concludes that LS test performance remained steady over decades, while COD ability declined with sex-based variations despite participants' early age. Our results offer crucial data for interventions to improve children's physical fitness: in particular for COD performance. PE teachers and coaches should prioritize improving COD over LS to improve these abilities and prevent physical fitness decline.

## Introduction

Most physical activities performed among adolescents and children include high-intensity tasks requiring the production of anaerobic energy. Linear speed (LS) and change of direction (COD) are part of those activities, where COD tasks are known for being more demanding in terms of motor and balance control (Giuriato et al., 2021a; Zając et al;., 2022). Moreover, LS and COD abilities are usually associated with enhanced performance in sports (Howley, 2001). They can be assessed using various maximal or submaximal field-based running tests. Generally, children prefer exercises that require the attainment of their full potential, yet for short time intervals. For these reasons, physical education (PE) teachers and coaches prefer 30–50 m trials for assessing running speed due to their simplicity, efficiency and reliability, whether conducted indoors or outdoors (Ortega et al., 2023).

During pre-adolescent and adolescent stages, general performance improves as a result of biological maturation, alongside an amelioration in spatiotemporal orientation skills and laterality, positively impacting both COD and LS abilities (Zouhal et al., 2018). Furthermore, tasks involving COD require greater movement of the body against gravity. Therefore, such tasks are more demanding for children with overweight or obesity, whose prevalence has been increasing over time (Morano et al., 2016). Indeed, D’Hondt et al. (2011) demonstrated a negative association between gross motor coordination and weight status that progressively increased with age from childhood to adolescence. Additionally, some studies linked COD and LS abilities to performance markers (Giuriato et al., 2021b; Gupta et al., 2022; Howley, 2001; Luberecka et al., 2022). For these reasons, temporal trends in LS and COD should therefore reflect corresponding trends in population fitness and health. Consequently, this may be the key to promoting healthy lifestyles based on physical activity (PA) levels among youngsters, leading to the development of appropriate public health policies.

In fact, there has been growing attention to secular trends or normative values about the physical fitness level of children and adolescents during growth (Ortega et al., 2023). In this context, many countries have provided a local description of how physical fitness changed across the years (Doyon et al., 2021; Hanssen-Doose et al., 2021; Lovecchio et al, 2019b). Also, some studies analyzed the physical fitness trends of Italian children and adolescents (Fiori et al., 2020; Giuriato et al., 2023a; Lovecchio et al., 2020) albeit with cardiorespiratory fitness as most evaluated (Lovecchio et al., 2022). However, our study considers another dimension of physical fitness, i.e., speed performance.

Indeed, LS and COD abilities are crucial predictors of team sports performance (Giuriato et al., 2021a, 2023a) and also important health markers (Howley, 2001). To the best of our knowledge, only two Italian papers have investigated the aforementioned abilities along 20 years or more (Lovecchio et al., 2022; Vandoni et al., 2022).

Due to the growing interest in the assessment of temporal trends and the limited data on LS and COD abilities trends, our study aimed to provide information about these abilities in Italian middle-school students. Moreover, the few data that described the trends of speed and COD performance revealed rather incohesive secular trends over the last 40 years (Matton et al., 2007; Tambalis et al., 2011; Tomkinson et al., 2007). Different test choices and/or assessment procedures to evaluate COD and LS skills in children and adolescents may partly explain those results. Additionally, several studies showed that COD was influenced by different factors, such as anthropometric characteristics, maturation, muscle qualities (Giuriato et al., 2023b), and participation in PA after school (Giuriato et al., 2021b).

Keeping this in perspective, the present analyses also attempted to “minimize” the effects of anthropometric characteristics and aimed to evaluate the real temporal trends in LS and COD abilities over 25 years. The purpose of this analysis was to examine how children’s abilities changed independently of the increase in fat mass that was recorded over the years.

Therefore, the main aim of this study was to provide an updated overview of speed abilities by estimating temporal trends in field-based COD and LS tests for Italian children over two decades, since the authors believe that these abilities had a lower decline compared to other nations’ trends (Matton et al., 2007) and to the cardiorespiratory fitness decline (Lovecchio and Zago, 2019). Three different speed tests (30-m, 60-m and 4 x 5-m shuttle run) were administered to evaluate the real temporal trend exclusive of anthropometric characteristics.

## Methods

### Participants

A total of 3884 Italian children/pre-adolescents (n = 2168 [56%] boys) aged 11–13 years were recruited across the period between 1990 and 2010, from a single middle-high school in northern Italy in a suburb-area near Milan (rural). Children of both sexes were recruited according to the following inclusion criteria: no-previously known neurological/orthopedic or cardiovascular diseases, no illness that could affect growth, and/or their active participation in school PE classes, healthy and eligible to perform PE activities (as certified upon medical examination), participation in physical education classes during the academic years.

Exclusion criteria were: orthopedic injuries during the last six months, any condition that would impede students from taking part in curricular PE classes, any medical condition that could hinder exercise participation. Parents or legal guardians read and signed written informed consent following a detailed description of the study procedures. Children expressed verbally their voluntary participation and were informed that they had the right to withdraw from the study at any point without repercussions. Participants did not receive any additional credit or benefit in return for their participation. This study was approved by the Ex-Irre Lombardia (National Agency for Scholastic development; Ministry of Instruction and Research; Prot. 1523; Cod 123.1/6; approval date: 27 December 1997) and was conducted in accordance with the Declaration of Helsinki (JAMA, 2013).

### Measures

Over the 20-year period, the same teacher assessed participants during two consecutive PE classes at the same time of the day (9:00 am to 1:00 pm) and in the first month of every school year (between mid-September and mid-October). To avoid nutritional problems, all participants had only breakfast before entering school facilities. All testing sessions were conducted in the same gym, with regulated and fixed temperature, following the civic rules, between 19 and 21°C.

Body mass and height were assessed according to the guidelines of the International Society for the Advancement of Kinanthropometry, i.e., with participants without shoes and while wearing light exercise clothes. Body mass was measured to the nearest 0.1 kg using a balance scale (Seca 864, Seca GmbH and Co., Hamburg, Germany), while body height was measured to the nearest 1 cm with a stadiometer (Seca 216, Seca GmbH and Co., Hamburg, Germany) and asking participants to stand upright with their head in the Frankfort plane. These variables were used to calculate the participants’ body mass index (BMI) using the following formula: body mass (kg) divided by squared body height (m^2^).

### Physical Fitness Tests

The assessment protocol consisted of a series of comprehensive physical fitness tests usually administrated in school settings (Artero et al., 2011; Lovecchio et al., 2019b). Field tests included in the assessment protocol are considered valid and reliable tools to evaluate the physical fitness level in children and adolescents (Artero et al., 2011). Furthermore, these tests are standardized, inexpensive in terms of equipment needed, and simple to administrate (Ruiz et al., 2011; Tomkinson and Olds, 2008).

### 30-m Speed Test

The participant performed a 30-m maximum speed test (ICC = 0.96) (Lovecchio et al., 2020). The start and end points were marked on the floor to ensure the correct distance measurement. The execution time was measured using a stopwatch (Stopwatch W073, SEIKO, Tokyo, Japan) having a time resolution of 0.01 s. The assessor, who was the same for all testing, started the stopwatch after the cue “3, 2, 1, go” and stopped the measurement when the participant crossed the end-line with one foot. Worse performances were identified by higher time scores. To ensure a correct task execution, the assessor showed the participants how to perform the test.

### 60-m Speed Test

The 60-m speed test was performed on an athletic track marked on the outer borders of the gymnasium’s floor. For this test, participants were asked to run within the marked lane at their maximal speed. The test preparation, as well as the data collection, was similar to the procedure described in the previous paragraph.

### 4 x 5-m Shuttle Run Test

The 4 x 5-m shuttle run test was used to evaluate participants' COD ability. During this test, participants had to run back and forth four times along a 5-m track at the highest possible speed (shuttle run). The turn angle was 180° for every COD for a total of three shuttles (ICC = 0.90; 95% CI 0.75–0.97) (Artero et al., 2011; Ortega et al., 2008). This test was used to investigate COD ability (Giuriato et al., 2021a) using the same stop-watch as indicated before. Every participant performed the test twice, however, only the better result was registered for further analysis. A higher execution time indicated a worse performance. To ensure a correct task execution, the assessor showed participants how to perform the test. The floor was checked after every participant to ensure a slip-proof floor condition.

### Statistical Analysis

**Figure 1:**
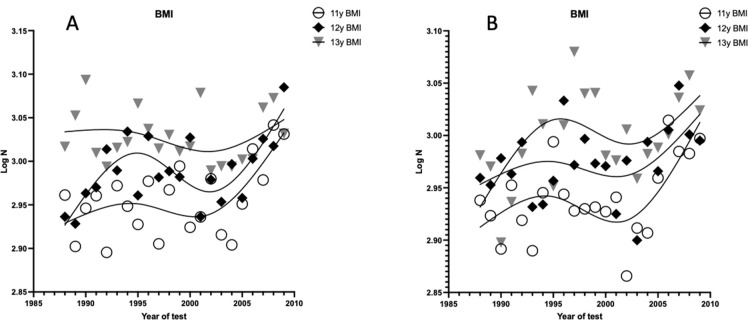
Temporal trends in lnBMI for northern Italian children aged 11–13 years between 1984 and 2010. Note: A = Girls; B = Boys

All data were manually entered into a spreadsheet and were checked for transcription errors, making corrections where appropriate. The dataset was also controlled for outliers where the execution time was close to zero and/or over twice the time of test-specific average performance (4 x 5 m, 30 m, 60 m). Similarly to other studies (Hanssen-Doose et al., 2021; Lovecchio et al., 2022), we first pooled the data into three-year study waves (e.g., 1990–92, 1993–95, … 2008–10) and then stratified our analysis into test-sex-age-specific groups (e.g., 11-year-old boys tested on the 4 x 5 m). Firstly, we calculated the natural logarithm (ln) of the body mass index (BMI) and represented it graphically to describe its changes over the years. Figure 1 illustrates this temporal trend for children aged 11–13 years between 1990 and 2010, showing an increase in the BMI over time.

For this reason, we executed a statistical analysis that considered the real trend of performance, adjusting the trends for anthropometric characteristics. The overall BMI-adjusted temporal trends for 4 x 5-m shuttle run, 30-m and 60-m speed tests were calculated using a weighted linear regression for each test-sex-age group as well as the corresponding 95% confidence interval (CI, obtained by multiplying the standard error of the regression coefficient per 1.96 (Tomkinson et al., 2021)). All temporal trends were expressed in three standard metrics: (a) as absolute trends (i.e., the regression coefficient), (b) as percent trends (i.e., the regression coefficient was expressed as a percentage of the mean for all the values of the regression), and (c) as standardized (Cohen’s) effect sizes (ESs) (i.e., the regression coefficient divided by the SD for all values of the regression). ESs of 0.2, 0.5, and 0.8 were respectively used as thresholds for small, moderate, and large ES, with ES <0.2 considered negligible (Cohen, 1988). Positive temporal trends indicated an increased time to complete the task, consequently outlining a declined performance, while negative trends showed a decreased task execution time, hence indicating an improvement in performance. We also calculated weighted mean trends for boys, girls, and all children of both sexes by pooling the test-sex-age-specific trends using a post-stratification population weighting procedure. This helped adjust for sampling bias by incorporating the underlying population demographics. Population weights were obtained from the United Nations sex-age-specific population estimates for Italy in 2000 (United Nations, 2019). All analyses were performed in SPSS Statistics (v14, IBM, Chicago, IL, USA).

## Results

### Performance Trends in the 4 x 5-m Shuttle Run Test

A significant large increase was observed in the mean 4 x 5-m shuttle run time (trend [95%CI]: 0.69 s [0.74, 0.64]; 9.15% [9.80, 8.50]; 1.36 ES [1.46, 1.26]). Sex stratified analyses revealed large increases in execution time for boys (1.01 ES [1.13, 0.90]) and girls (1.66 ES [1.82, 1.50]).

BMI-adjusted 4 x 5-m analyses showed that temporal trends improved with age, in particular the decline rate was slowing down with age in girls, while boys showed an increase (11 compared to 13 years) after a slow decrease (12 years).

### Performance Trends in the 30-m Speed Test

With regard to 30-m speed test performance, analyses revealed negligible trends in the BMI-adjusted mean (0.00 s [0.03, −0.03]; 0.01% [0.63, −0.62]; -0.01 ES [0.06, −0.07]) that were confirmed when stratified for boys (0.00 ES [0.04, −0.04]) and girls (0.00 ES [0.05, −0.05]).

### Performance Trends in the 60-m Speed Test

Considering the BMI-adjusted mean, negligible trends were found in 60-m speed test performance (−0.10 s [−0.01, −0.11]; −0.90% [0.03, −1.83]; −0.07 ES [−0.01, −0.07]). In particular, negligible and small trends were found for boys (0.10 ES [0.26, −0.04]) and girls (−0.21 ES [0.04, −0.34]), respectively.

## Discussion

The present investigation provides an update on age and sex-specific BMI-weighted LS and COD trends of Italian children. Our results demonstrate: i) a large increase in 4 x 5-m shuttle run time for both boys and girls according to BMI-adjusted analyses, showing a slow decline with age in girls and an increase (11 compared to 13 years), after a slow decline (12 years), in boys; ii) a negligible trend in mean BMI-adjusted 30-m speed performance, denoting small changes over time when stratified for boys and girls, and iii) no difference in 60-m speed test performance between successive years according to BMI-adjusted mean values. The main objective of this study was to compare LS (30-m, 60-m test) and COD (4 x 5-m) performance among Northern Italian children aged between 11 and 13 years over a time span of

20 years (from 1990 to 2010). According to the current analysis, independently of the weighing variable (BMI) a decrease in performance over time was observed only in 4 x 5-m shuttle run performance for both girls and boys and in every age group. The stagnation of 30-m and 60-m speed test performance is in accordance with what was reported by Eberhardt et al. (2020), showing no differences over time in 30-m speed test performance. Moreover, Lovecchio et al. (2020) showed non-significant changes in speed test performance (the same 30-m speed test) from 2004 to 2013 among Italian children (boys 5.18 + 0.46 s; girls 5.33 + 0.44 s). Similar findings were reported by Hanssen-Doose et al. (2021) who initially recorded a mild increase in physical fitness levels of German children and adolescents when comparing baseline results (2003–2006) to wave 1 (2009–2012), however, their physical fitness levels remained the same between wave 1 and wave 2 (2014–2017). Another study (Rosa et al., 2023) indicated that physical fitness levels of Portuguese children did not differ between 2008 and 2018.

**Table 1:** Descriptive statistics for 4 x 5-m shuttle run, 30-m and 60-m sprint tests among northern Italian boys aged 11–13 years between 1984 and 2010.

Boys							
			11		12		13
	Years wave	n	Mean + SD	n	Mean + SD	n	Mean + SD
**4 x 5-m shuttle run test (s)**	1984–86	53	7.38 + 0.39	60	7.27 + 0.73	38	7.02 + 0.36
	1987–89	69	7.32 + 0.41	94	7.12 + 0.38	129	6.88 + 0.45
	1990–92	79	7.34 + 0.50	70	7.02 + 0.39	58	6.87 + 0.36
	1993–95	74	7.31 + 0.42	66	7.09 + 0.59	64	6.9 + 0.45
	1996–98	85	7.44 + 0.50	64	7.25 + 0.41	82	6.90 + 0.37
	1999–01	92	7.53 + 0.51	72	7.31 + 0.52	75	7.11 + 0.51
	2002–04	72	7.43 + 0.48	81	7.29 + 0.52	73	6.96 + 0.44
	2005–07	110	7.66 + 0.60	105	7.50 + 0.61	91	7.22 + 0.54
	2008–09	77	7.98 + 0.67	81	7.64 + 0.59	65	7.53 + 0.61
**30-m sprint test (s)**	1984–86	193	5.36 + 0.34	194	5.15 + 0.36	194	4.94 + 0.40
	1987–89	66	5.32 + 0.37	104	5.11 + 0.39	130	4.87 + 0.37
	1990–92	81	5.39 + 0.38	76	5.15 + 0.41	56	4.92 + 0.41
	1993–95	80	5.35 + 0.37	74	5.13 + 0.56	68	5.05 + 0.60
	1996–98	87	5.40 + 0.45	78	5.28 + 0.42	83	4.93 + 0.44
	1999–01	91	5.32 + 0.41	71	5.12 + 0.40	76	4.94 + 0.45
	2002–04	70	5.26 + 0.32	65	5.09 + 0.43	71	4.83 + 0.41
	2005–07	111	5.39 + 0.48	103	5.16 + 0.56	98	4.85 + 0.53
	2008–09	74	5.31 + 0.56	79	5.26 + 0.60	67	5.01 + 0.51
**60-m sprint test (s)**	1984–86	193	10.33 + 0.74	194	9.93 + 0.82	192	9.50 + 0.93
	1987–89	66	10.34 + 0.82	104	9.87 + 0.95	130	9.29 + 0.84
	1990–92	82	10.58 + 0.89	76	10.01 + 0.93	56	9.60 + 0.92
	1993–95	80	10.32 + 0.82	74	10.07 + 1.78	66	9.62 + 1.31
	1996–98	87	10.67 + 1.15	78	10.31 + 0.97	81	9.54 + 1.04
	1999–01	90	10.35 + 0.98	71	9.90 + 0.98	75	9.48 + 0.95
	2002–04	67	10.31 + 0.84	65	9.90 + 0.86	67	9.32 + 0.85
	2005–07	111	10.51 + 1.02	103	10.04 + 1.24	98	9.31 + 1.19
	2008–09	74	10.46 + 1.18	79	10.18 + 1.26	67	9.69 + 1.07

**Table 2:** Descriptive statistics for 4 x 5-m shuttle run, 30-m and 60-m sprint tests among northern Italian girls aged 11–13 years between 1987 and 2010.

Girls								
			11		12		13
	Year wave	n	Mean + SD	n	Mean + SD	n	Mean + SD
**4 x 5-m shuttle run test (s)**	1984–86	28	7.48 + 0.40	29	7.25 + 0.27	37	7.24 + 0.33
	1987–89	77	7.54 + 0.43	63	7.38 + 0.39	78	7.25 + 0.39
	1990–92	62	7.66 + 0.46	74	7.43 + 0.42	95	7.33 + 0.49
	1993–95	64	7.60 + 0.37	52	7.60 + 0.46	75	7.34 + 0.46
	1996–98	90	7.94 + 0.58	70	7.71 + 0.67	91	7.40 + 0.46
	1999–01	70	7.78 + 0.55	95	7.54 + 0.51	80	7.53 + 0.62
	2002–04	90	7.91 + 0.56	88	7.85 + 0.66	78	7.62 + 0.54
	2005–07	88	8.27 + 0.52	68	8.00 + 0.58	74	7.89 + 0.53
**30-m sprint test (s)**	2008–09	28	5.49 + 0.43	30	5.29 + 0.26	38	5.30 + 0.31
	1984–86	79	5.44 + 0.37	74	5.27 + 0.35	79	5.15 + 0.33
	1987–89	65	5.62 + 0.48	82	5.36 + 0.45	96	5.19 + 0.52
	1990–92	59	5.45 + 0.40	60	5.38 + 0.41	71	5.30 + 0.44
	1993–95	84	5.58 + 0.49	67	5.29 + 0.36	92	5.21 + 0.38
	1996–98	65	5.47 + 0.44	78	5.25 + 0.42	69	5.36 + 0.47
	1999–01	86	5.41 + 0.39	91	5.27 + 0.39	85	5.15 + 0.34
	2002–04	85	5.57 + 0.45	63	5.41 + 0.43	72	5.25 + 0.41
**60-m sprint test (s)**	2005–07	28	11.20 + 1.61	30	10.29 + 0.65	38	10.29 + 0.69
	2008–09	79	10.74 + 0.87	73	10.25 + 0.88	79	10.28 + 1.33
	1984–86	66	11.21 + 1.38	82	10.48 + 1.09	96	10.12 + 1.14
	1987–89	59	10.76 + 0.77	60	10.45 + 0.88	69	10.45 + 1.48
	1990–92	84	10.95 + 1.20	68	10.41 + 0.90	92	10.11 + 0.86
	1993–95	61	10.76 + 0.99	75	10.20 + 0.95	65	10.51 + 1.16
	1996–98	86	10.59 + 0.82	91	10.30 + 0.81	85	9.96 + 0.86
	1999–01	85	10.87 + 0.97	63	10.56 + 1.02	72	10.07 + 0.86

**Table 3:** Temporal trends in means for BMI-adjusted 4 x 5-m shuttle run time among northern Italian children aged 11–13 years between 1984 and 2010.

				Trends in means (95% CI)		
	Gender	Age	N	Absolute	Percent	Standardized ES
**BMI-adjusted 4 x 5-m (s)**	Boys	11	711	0.57 (0.59–0.55)	7.57 (7.62–7.52)	1.05 (1.07–1.03)
	Boys	12	693	0.50 (0.51–0.49)	6.89 (6.94–6.84)	0.88 (0.90–0.86)
	Boys	13	764	0.56 (0.57–0.55)	7.78 (7.83–7.73)	1.11 (1.13–1.09)
	Girls	11	569	0.92 (0.94–0.90)	11.79 (11.84–11.74)	1.89 (1.91–0.87)
	Girls	12	539	0.76 (0.78–0.74)	10.02 (10.04–10.00)	1.50 (1.52–1.48)
	Girls	13	608	0.77 (0.78–0.76)	10.32 (10.33–10.31)	1.60 (1.62–1.58)

Notes: Positive trends indicated increases in time to perform the test (temporal declines in performance) and negative trends indicated declines in time to perform the test (temporal increases in performance)

**Table 4:** Temporal trends in means for BMI-adjusted 30-m sprint test time among northern Italian children aged 11–13 years between 1984 and 2010.

				Trends in means (95% CI)		
	Gender	Age	N	Absolute	Percent	Standardized ES
**BMI-adjusted 4 x 5 m (s)**	Boys	11	711	0.57 (0.59–0.55)	7.57 (7.62–7.52)	1.05 (1.07–1.03)
	Boys	12	693	0.50 (0.51–0.49)	6.89 (6.94–6.84)	0.88 (0.90–0.86)
	Boys	13	764	0.56 (0.57–0.55)	7.78 (7.83–7.73)	1.11 (1.13–1.09)
	Girls	11	569	0.92 (0.94–0.90)	11.79 (11.84–11.74)	1.89 (1.91–0.87)
	Girls	12	539	0.76 (0.78–0.74)	10.02 (10.04–10.00)	1.50 (1.52–1.48)
	Girls	13	608	0.77 (0.78–0.76)	10.32 (10.33–10.31)	1.60 (1.62–1.58)

Notes: Positive trends indicated increases in time to perform the test (temporal declines in performance) and negative trends indicated declines in time to perform the test (temporal increases in performance)

**Table 5:** Temporal trends in means for BMI-adjusted 60-m sprint test time among northern Italian children aged 11–13 years between 1984 and 2010.

				Trends in means (95% CI)		
	Gender	Age	N	Absolute	Percent	Standardized ES
**BMI-adjusted 60 m (s)**	Boys	11	850	0.07 (0.08–0.06)	0.70 (0.85–0.55)	0.18 (0.19–0.17)
	Boys	12	842	0.13 (0.14–0.12)	1.35 (1.37–1.33)	0.14 (0.15–0.13)
	Boys	13	832	−0.03 (−0.03–−0.03)	−0.32 (−0.33–0.31)	−0.03 (−0.03–−0.03)
	Girls	11	549	−0.49 (−0.50–−0.51)	−4.56 (−4.60–−4.52)	−0.44 (−0.45–−0.43)
	Girls	12	542	0.01 (0.01–0.01)	0.95 (0.97–0.93)	0.01 (0.01–0.01)
	Girls	13	602	−0.21 (−0.22–−0.20)	−2.07 (−2.09–−2.05)	−0.20 (−0.21–−0.19)

Notes: Positive trends indicated increases in time to perform the test (temporal declines in performance) and negative trends indicated declines in time to perform the test (temporal increases in performance)

The results of the 4 x 5-m shuttle run test underline a decrease in nonlinear speed performance across years (Matton et al., 2007; Tomkinson and Olds, 2007; Vandoni et al., 2022). Similar findings were obtained by Matton et al. (2007) that showed a decrease in 10 x 5-m shuttle run test performance over a time span of thirty-five years. Also Tambalis et al. (2011) showed a decrease in COD performance in Greek children from 1997 to 2007 which was coupled with an increase in the obesity status of this population. Despite the above-mentioned studies which are in accordance with our results, others showed stable or increased COD performances (Moliner-Urdiales et al., 2010), indicating unclear temporal trends with regard to COD ability. However, it should be acknowledged that the cited studies did not take into account participants’ anthropometric characteristics in their analyses, showing an influenced temporal trend. This gap raises a significant concern, as different BMI values of children could potentially explain the contrasting trends observed in these studies. Indeed, anthropometric measures are an important indicator of physical fitness (Gatti et al., 2023; Lovecchio and Zago, 2019). Basterfield et al. (2022) reported how children’s BMI and 20-m shuttle run test performance changed adversely over one year from October 2019 to November 2020, demonstrating the influence of the BMI on performance.

On the other hand, the difference in trends related to 4 x 5-m shuttle run and LS tests may be associated with an increase in sedentary behaviors along with a reduction in PA levels that have been recorded worldwide (Currie et al., 2012). In fact, organized PA could be the first performance predictor in the 4 x 5-m shuttle run test, showing that this type of activity is fundamental for the adequate development of motor abilities while growing (Morano et al., 2016). Probably non-linear tests, as is the 4 x 5-m shuttle run task, may be more affected by a decrease in PA levels (Guthold et al., 2018) when compared to linear speed tests such as the 30-m and 60-m tests. Indeed, 30-m and 60-m linear speed requires less motor coordination and balance control. In fact, coordination and PA are strictly related: some studies showed strong evidence for a positive association between motor coordination and the amount of PA practice (Giuriato et al., 2021b), suggesting that a reduced PA level together with an increased sedentary behavior may negatively influence 4 x 5-m shuttle run test performance over the years. Another possible explanation for the different results found in the 4 x 5-m shuttle run and the LS test could be related to different requirements in terms of coordination and effort levels. From a physiological point of view, the 4 x 5-m shuttle run test requires a mixture of physical abilities (Sheppard and Young, 2006), including balance, strength, and power, as noted by McBurnie and Dos’Santos (2022), which are different from physiological demands of LS tests.

The strengths of this study include the large sample size and the use of valid and reliable tests (4 x 5-m shuttle run, 30-m and 60-m speed tests) to assess LS and COD abilities in adolescents (Carnevale Pellino et al., 2020). Additionally, this research model could be employed to investigate LS and COD abilities of other populations presenting different networks of social, behavioral, psychosocial, and psychological factors.

The current study also displays some limitations. Firstly, the lack of body composition information did not allow an in-depth data analysis limiting our results to the use of BMI-adjusted variables. Secondly, the sample size was only composed of Caucasian participants recruited from one school in northern Italy, therefore the results may not apply to other demographic groups or countries. Another possible limitation is the lack of detailed information about children’s PA levels. However, it is important to note that the IPAQ questionnaire, which is commonly used to evaluate PA levels, was developed between 1997 and 1998 (Craig et al., 2003), while the Italian version was validated only in 2010 (Mannocci et al., 2010). Therefore, it was impossible to evaluate PA levels before that year. Finally, even though our data include 25 years of evaluation, they only extend up to 2009. As a result, they may not accurately reflect current performance of middle-school students. Therefore, further collaboration among coaches and PE teachers is crucial to update LS and COD trends in the last decade, especially after the COVID-19 restrictions.

## Conclusions

In conclusion, our results show that performance levels in LS tests did not decline over decades, while COD ability, assessed with the 4 x 5-m shuttle run test, revealed a decline in performance that varied according to participants’ sex despite their early age. Furthermore, the current results provide objective data on physical performance, which is crucial for changing knowledge based on a subjective mindset. Also, such data lead to an in-depth understanding of the specific activities in which we can intervene effectively to improve physical fitness, and consequently, children’s health status. Moreover, since the 4 x 5-m shuttle run test is an ecological test that simulates a sport-specific scenario, PE teachers and coaches should take its performance-related decline into account. They should consider spending some of their limited teaching and coaching hours strategically, focusing on improving COD ability rather than LS to prevent further decline in performance and physical fitness levels.
